# Supplementary material for: A Novel 4-gene Score to Predict Survival, Distant Metastasis and Response to Neoadjuvant Therapy in Breast Cancer
Source: Cancers (Basel). 2020 May 2;12(5):1148. doi: 10.3390/cancers12051148 (PMC7281399; doi:10.3390/cancers12051148)
Supplement: Supplementary file 1 [file cancers-12-01148-s001.pdf]

cancers

MDPI

Supplementary Materials

A Novel 4-gene Score to Predict Survival, Distant Metastasis, and Response to Neoadjuvant Therapy in Breast Cancer

Masanori Oshi, Eriko Katsuta, Li Yan, John M.L. Ebos, Omar M. Rashid, Ryusei Matsuyama, Itaru Endo and Kazuaki Takabe

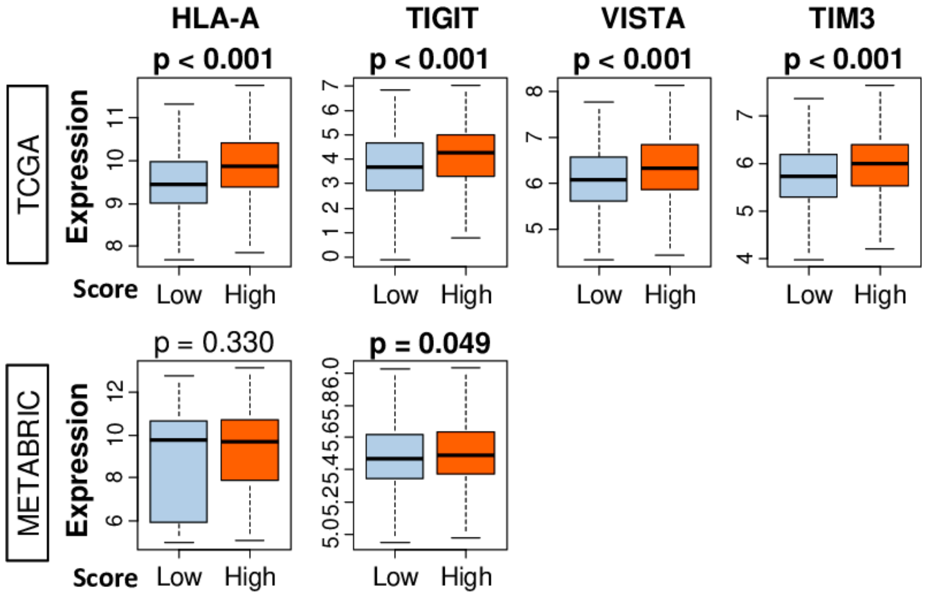

Figure S1. Comparisons of gene expression (log_2 transcripts per million) of immune response genes in tumors of high or low 4-gene scores of TCGA and METABRIC cohorts. Boxplots of expression of immune response genes in tumors with high and low 4-gene scores. Within-cohort first tertile value of the 4-gene score was used to classify tumors into low and high groups. All boxplots are of Tukey type and the boxes depict medians and inter-quartile ranges. The depicted p values were calculated with standard t tests. TIM3 and VISTA gene expression data were unavailable for the METABRIC cohort. HLA, human leukocyte antigen; TIGIT, tyrosine-based inhibitory motif domain; TIM3, T cell immunoglobulin domain and mucin domain 3; VISTA, V-domain immunoglobulin-containing suppressor of T cell activation.

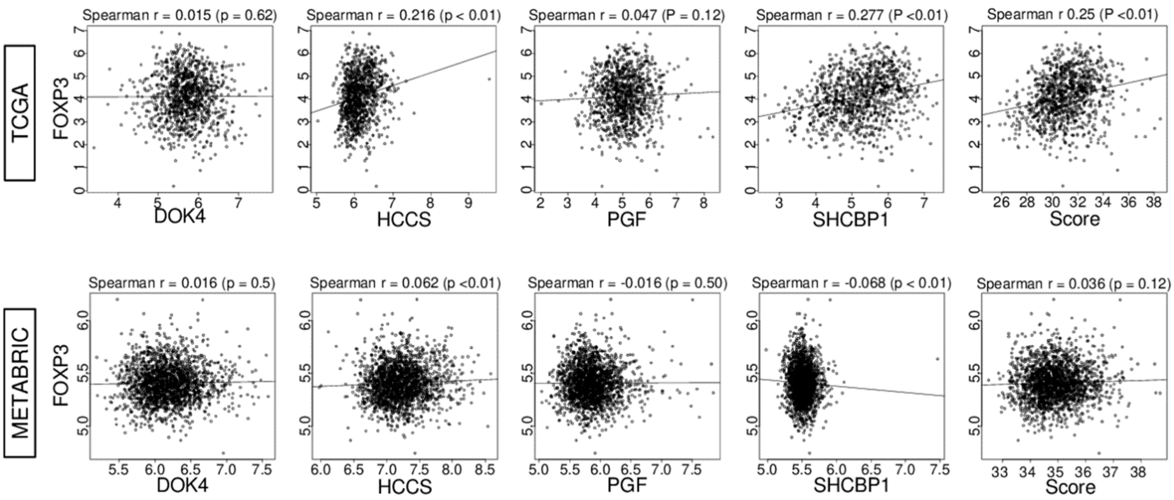

Figure S2. Correlation plots of FOXP3 gene expression with the 4-gene score and expression levels of its constituent genes in tumors of TCGA and METABRIC cohorts. Least squares regression lines, and correlation coefficient (r) and p values in Spearman tests are shown.

© 2020 by the authors. Licensee MDPI, Basel, Switzerland. This article is an open access article distributed under the terms and conditions of the Creative Commons Attribution (CC BY) license (http://creativecommons.org/licenses/by/4.0/).
